# Smart portable rehabilitation devices

**DOI:** 10.1186/1743-0003-2-18

**Published:** 2005-07-12

**Authors:** Constantinos Mavroidis, Jason Nikitczuk, Brian Weinberg, Gil Danaher, Katherine Jensen, Philip Pelletier, Jennifer Prugnarola, Ryan Stuart, Roberto Arango, Matt Leahey, Robert Pavone, Andrew Provo, Dan Yasevac

**Affiliations:** 1 Department of Mechanical & Industrial EngineeringNortheastern University360 Huntington Avenue, Boston MA 02115, USA

## Abstract

**Background:**

The majority of current portable orthotic devices and rehabilitative braces provide stability, apply precise pressure, or help maintain alignment of the joints with out the capability for real time monitoring of the patient's motions and forces and without the ability for real time adjustments of the applied forces and motions. Improved technology has allowed for advancements where these devices can be designed to apply a form of tension to resist motion of the joint. These devices induce quicker recovery and are more effective at restoring proper biomechanics and improving muscle function. However, their shortcoming is in their inability to be adjusted in real-time, which is the most ideal form of a device for rehabilitation. This introduces a second class of devices beyond passive orthotics. It is comprised of "active" or powered devices, and although more complicated in design, they are definitely the most versatile. An active or powered orthotic, usually employs some type of actuator(s).

**Methods:**

In this paper we present several new advancements in the area of smart rehabilitation devices that have been developed by the Northeastern University Robotics and Mechatronics Laboratory. They are all compact, wearable and portable devices and boast re-programmable, real time computer controlled functions as the central theme behind their operation. The sensory information and computer control of the three described devices make for highly efficient and versatile systems that represent a whole new breed in wearable rehabilitation devices. Their applications range from active-assistive rehabilitation to resistance exercise and even have applications in gait training. The three devices described are: a transportable continuous passive motion elbow device, a wearable electro-rheological fluid based knee resistance device, and a wearable electrical stimulation and biofeedback knee device.

**Results:**

Laboratory tests of the devices demonstrated that they were able to meet their design objectives. The prototypes of portable rehabilitation devices presented here did demonstrate that these concepts are capable of the performance their commercially available but non-portable counterparts exhibit.

**Conclusion:**

Smart, portable devices with the ability for real time monitoring and adjustment open a new era in rehabilitation where the recovery process could be dramatically improved.

## Introduction

During the last several decades a great deal of work has been undertaken for developing devices to accelerate recovery from injuries, operations and other complications. Many successful devices and methods have come out of this work. This included a general division of the recovery process into several phases.

In the early stages of therapy, passive rehabilitation is often a preferred method for reducing swelling, alleviating pain, and restoring range of motion. This consists of moving the limb with the muscles remaining passive and often involves devices such as Continuous Passive Motion (CPM) machines. The next stage of rehabilitation is often an active-assistive movement phase, which involves using external assistance to assist the muscles in moving the joint in order to reestablish neuromuscular control. Various different methods are presently used for this purpose, including various braces, orthoses, and large machines. The final stages aim at returning an individual to normal activities via resistance exercises that are usually focused at regaining muscle strength. Isokinetic machines are well known, ideally suited systems for achieving this final goal.

In this paper we present a compilation of several new developments in the area of portable and smart rehabilitation devices, being developed by the Northeastern University Robotics and Mechatronics Laboratory. The devices that will be presented in this paper are:

a) a transportable continuous passive motion device ideally suited for nearly any aspect of the earlier rehabilitation stages of the elbow,

b) an electro-rheological fluid based device for resistance exercises and control of the knee;

c) an electrical stimulation and biofeedback device for active-assistive exercises of the knee.

The presented devices span across all three of the mentioned phases in rehabilitation and exhibit many advantages over current technology. All three devices have been developed to increase the efficiency in rehabilitation exercises while remaining compact and portable. In each case, the capabilities of present technology have been taken into consideration and each device is designed to have similar characteristics. The most notable difference however, between this new breed of rehabilitation devices and currently used equipment is their highly adaptive, versatile and reprogrammable nature. Computer control is intrinsic to the design of each device presented in this paper and is a central theme behind their operation. This makes for highly effective tools for a wide range of applications. More specifically, the advantages of our advanced orthotics can be divided into four main categories: cost, portability, real-time abilities, and versatility.

### Cost

The designed advanced rehabilitation devices resolve several issues with cost with present-day technology. Every initial feasibility prototype fell just short of $2,000 to build. With all the electrical and sensory components that need to be added to each device for a final functional and marketable product, it is estimated to cost approximately $,3500. A state of the art, computer controlled Isokinetic Machine, such as the Biodex System 3 Pro, can be bought for over $40,000. Clearly, direct cost comparisons warrant the use of advanced rehabilitation devices over the comparable rehabilitation machines. Indirectly, the smaller size of the advanced rehabilitation devices also brings down costs by eliminating concerns with storage, portability, and weight. Rehabilitation machines are inherently large and require a permanent or semi-permanent set-up. The facility able to house such a device along with the personnel required for operating them is at a large economical disadvantage to smaller facilities using these much more compact advanced rehabilitation devices. Numerous devices, at an overall lower cost than a single machine, could be stored in something as simple as a closet. The devices themselves could easily be transported by the patients for use at home as well, saving time and money in the costly trips to specialized facilities.

### Portability

The most important feature of such a device is the fact that it is a portable and wearable form of rehabilitation. The compact and lightweight characteristics of these advanced rehabilitation devices allow them to be used in an average chair, while standing, or perhaps even during ambulatory motion. Their application is limited by only the user's abilities, meaning weaker patients can use it for resistive exercises while stronger patients can use it for both weight training as well as proper gait training. Equally noteworthy is the new capability for patients to take the device with them and exercise on their own time, from the comfort of their own home or office, or for use during their every day routines. All exercises being recorded, a physical therapist could simply download the data remotely and analyze the effectiveness and efficiency of the device, without ever needing the patient to revisit the medical facility.

### Real-Time Abilities

The ability of rehabilitation machines to function in real-time, is what separates them from their less efficient counterparts, the conventional orthotics. The inclusion of this feature is intrinsic to the utilization of compact advanced actuators and smart sensors in our portable and smart rehabilitation devices. They are easily computer controlled, and can react in the order of milliseconds. With such controllability, a rehabilitation regime can be perfectly tailored to each patient's individual needs very easily. Ideally, with closed loop control, feedback from the sensors would allow a computer to calculate the efficiency of each specific exercise and alter them in real-time accordingly to achieve optimal levels of rehabilitation.

### Versatility

Probably the most unique advantage of these devices arises from their versatility. With comparable abilities to modern day rehabilitation machines and similar functionality to several different types of these machines, the all-encompassing nature of these advanced orthotics alone makes them equally as versatile. However, due to all their additional strengths and advantages, including size, portability, and real-time computer control, the applications of these devices goes above and beyond those of the competing technologies. In the area of rehabilitation, these advanced orthotics could be a valuable tool in the development of new rehabilitation exercises and regimes. With complete control and tunability of the device, any type of complex algorithm defining the motion or resistance of the patient's knee could be easily implemented. Whole new concepts in rehabilitation or weight training could potentially be developed using this device as a research instrument, providing all the force and feedback necessary for any type of investigation. For more complicated medical disabilities, for instance in the case of gait correction in stroke patients, both analysis and implementation of newly developed methods could also be easily performed. Other potential applications, showing the extreme versatility of this device, include virtual reality simulations and athletic training, such as in rowing and weight-lifting.

## Portable continuous passive motion elbow device

### Overview

A transportable elbow rehabilitation device for use throughout the entire process of rehabilitating patient's with severe elbow trauma was designed, built, tested and optimized. The apparatus has three settings – passive, active and bracing. The device consists of a D.C. motor, gearbox, encoder, clutch and brake located in a portable unit, attached through a flexible shaft to an absolute encoder located on an elbow brace. In the passive setting, the device moves the forearm about the elbow joint to regain the range of motion. It acts as a "smart" continuous passive motion machine because constant sensor feedback enables the device to push to the patient's maximum range of motion during each cycle. Torque and speed of the passive movement is controlled through the current and voltage, respectively, drawn by the motor. In the active setting, variable resistance is applied using the brake. Both settings are controlled, monitored and recorded using a LabVIEW program on a personal computer, with specific protocol defined by a physician, physical therapist or athletic trainer. Currently available CPM machines are not transportable, do not sense the patient's range of motion and do not allow for an active setting. By combining three different functions (active mode, passive mode and bracing) of the device into one transportable unit, the next generation of elbow rehabilitation devices was created.

### Significance and Background

Following surgery, stroke or other injury to the elbow, a patient's range of motion is reduced due to trauma experienced at that location. Increasing the user's range of motion is the first step in a full recovery. This is accomplished through passive motion, where the patient's forearm is actively forced to flex and extend, followed by strength training. At this point, most doctors or physical therapists begin to use a continuous passive motion (CPM) machine. A CPM machine moves the forearm about the elbow joint to regain the patient's range of motion. Unfortunately, current CPM machines often involve a complicated set up, are non-portable, and are most importantly inefficient. Their inefficiency arises from their inability to recognize when the user's range of motion has increased. The machine must be continuously monitored and manually reset to further increase the range of motion. A related concern is the possibility of forcing the patient's arm past his or her range of motion resulting in further damage to the joint. The range of motion can only be increased in very small increments and movement about the elbow is nonproductive once the preset range is achieved. There are several patents covering the range of elbow rehabilitation devices [1-6]. Several companies such as Breg, Dyna Splint, Ultra Flex, Biodex CPM and the Bledsoe Extender Arm Brace have products out on the market that immobilize the injury and prepare the elbow for rehabilitation [7-12]. However, the only portable devices that are available provide either spring tension against an elbow contracture to achieve increased motion or locking mechanisms to restrict motion and prevent further injury. There are currently no commercially available devices that are portable and provide the passive motion required in the beginning stages of elbow rehabilitation.

### Design and Prototype

A wearable and portable CPM device that senses increases in the patient's range of motion and simultaneously increases its range of motion has been developed in our laboratory. The patient's torque and motion limits are inputted into a computer interface. The program then monitors and controls all of the components of the device, progressively increasing the user's range of motion about the joint within the torque and motion range. Through sensory input, the computer senses when the user's muscular resistance has reached its limit and signals a reversal in direction of motion, allowing for maximum range of motion to be reached quickly and efficiently, without harm to the patient.

This new transportable elbow rehabilitation device also safely and efficiently assists throughout the rest of the entire rehabilitation process, including bracing the joint and building muscle mass. The device has adjustable settings for each stage of rehabilitation. The passive motion setting, as mentioned, uses constant sensor feedback that enables the device to progressively increase the user's range of motion. The device is also capable of applying variable resistance about the elbow joint to build muscle mass once the patient's ideal range of motion has been achieved. This mode is very similar to Isokinetic machines. Finally, the device is also capable of acting as a simple brace: either locking in place to prevent the user from moving his or her arm, or disengaging entirely to provide mediolateral support. The combination of all three modes with adjustable settings within each mode, allows this device to be utilized through the entire rehabilitation process for a variety of elbow injuries.

**Figure 1:**
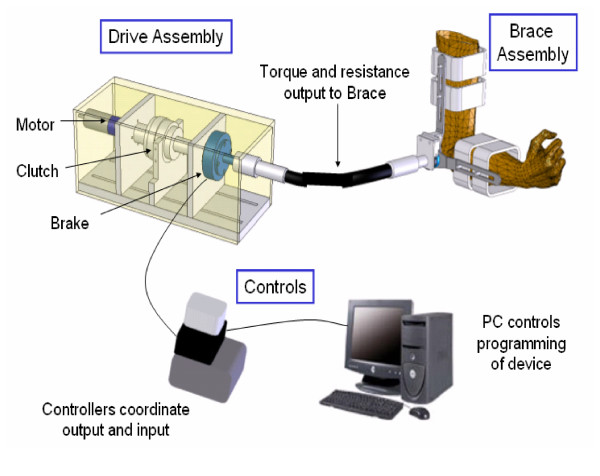
CAD rendering of portable elbow device.

The elbow device is lightweight, easily programmable and transportable. A CAD rendering of the device and its components can be seen in Figure 1.

The device can be split into two subsystems. The first is the brace worn by the patient. It is designed around an Orthomerica Prime Elbow System brace and includes an optical encoder, for measurements of position and velocity and an attachment point for a flexible shaft. This flexible shaft connects the brace to the second subsystem, a tabletop drive assembly unit that provides the functionality of the device. It houses a DC motor, an electrically controlled clutch and magneto-resistive fluid brake and is designed to fit in a backpack. The flexible shaft allows the user to move freely while the device is in use and easily detaches from the brace, providing the patient with a protective elbow brace to continue daily routines when not in use.

The motor-gearbox combination provides the passive exercise motion for the patient to increase his or her range of motion. A current limiter set in the motor control box ensures that the patient does not exceed his or her range of motion. The current measurement is converted to torque resistance in the computer and once the preprogrammed limit is exceeded; the motor direction is reversed

Between the motor and flexible shaft is the electrically controlled clutch. It serves mainly as a safety feature for the patient. It disengages if the user hits the stop switch, if the current exceeds the motors limited levels, or when the active feature is in use. This active feature functions with the use of a magneto-resistive fluid (MRF) brake. The brake is manufactured by Lord Corporation and features a simple yet rugged design, high torque, and quiet operation. It provides smooth, controllable resistance to the patient for building muscle and tissue strength in the elbow joint. The MRF brake and motor in-line assembly can also be used in combination. This provides the user with an extra impulse of motion after they have used the resistive feature to their maximum range of motion or active-assistance.

**Figure 2:**
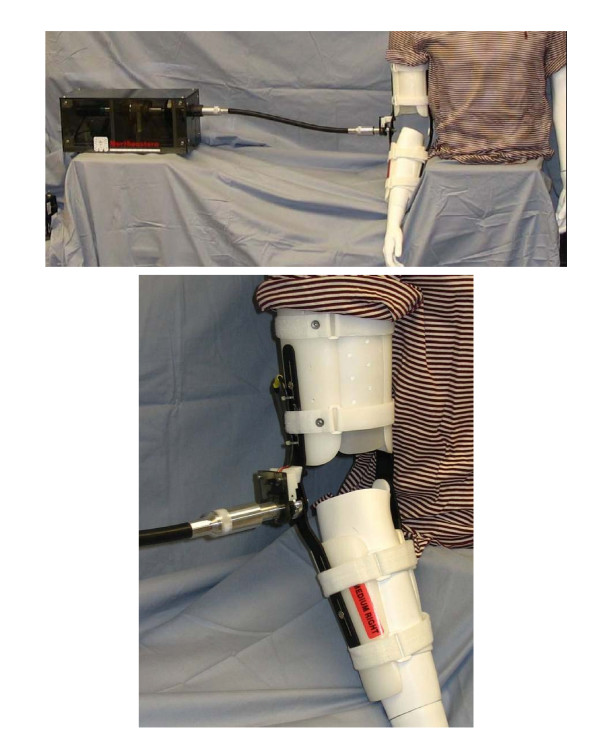
Portable elbow device: full assembly.

The device was constructed for feasibility analysis. Figure 2 shows the full assembly. The device has a mobility range of 155 degrees. The motor, gearbox (1:134 gear ratio), and clutch combination was found to be capable of producing 10 N·m of torque. The MR brake was found to have a maximum resistive torque capability of 5.6 N· m. All these performance characteristics can be found listed in Table 1.

**Table 1:** Elbow Device Design Summary

**System Characteristics:**	
Range of motion (0° being full extension)	± 77.5°
Continuous Passive Motion capabilities	10 N·m
Isokinetic capabilities	5.6 N·m

**Figure 3:**
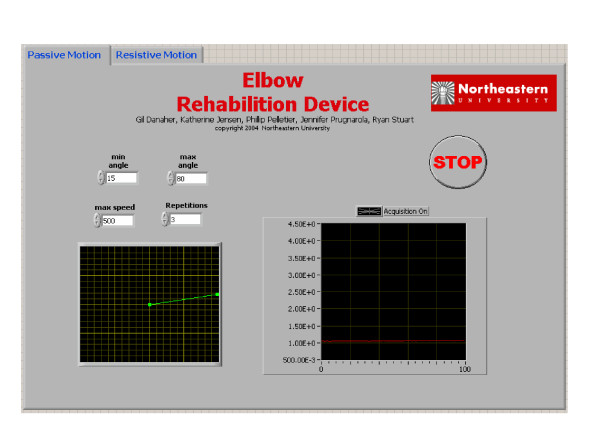
Passive motion graphical interface.

The motor, encoder, brake, and clutch are controlled through a LabVIEW 7.0 program on the PC. The user interface is simple, and utilizes tab controls that allow the user to select either the active or passive setting. In the active setting, the user inputs the resistive torque required for exercise and can see a real-time plot of the joints position and resistance level. Inputs in the passive setting include the number of repetitions, speed, and minimum and maximum angles. The user can view real time plots of position and torque being applied to their joint during the exercise routine. The graphic user interfaces for the passive motion can be seen in Figure 3.

## Electro-rheological fluid based knee resistance device

### Overview

This device aims to demonstrate the feasibility of using Electro-Rheological Fluid (ERF) actuators in orthotics, creating a new breed of rehabilitation devices. ERFs are fluids that experience dramatic changes in rheological properties, such as viscosity or yield stress, in the presence of an electric field. Using the electrically controlled rheological properties of ERFs, compact actuators with an ability to supply high resistive torques in a controllable and tunable fashion, have been developed. This study involves the design, fabrication and testing of an ERF based knee orthotic device and the innovative ERF actuators it uses. The knee orthotic is achieved through a standard brace design with a polycentric hinge and gear system. Coupled to this are two Flat-Plate ERF actuators, given that name for their characteristic set of parallel flat plates allowing for actuation of the fluid. The overall knee orthotic system is designed to resist up to 25.4% of an average human knee's torque abilities and be controlled in real-time. The goal of this work is to provide a much more efficient means of rehabilitation over the average orthotic, while matching the proficiency of rehabilitation machines, all in a smaller, simpler, and more cost efficient design.

### Significance and Background

An orthotic device by strict definition is a specialized mechanical device that supports or supplements weakened or abnormal joints or limbs. The majority of these devices can be categorized as passive, meaning the resistance or support they provide is not changed in real time. The Sports Medicine Committee of the American Academy of Orthopedic Surgeons has further classified these types of braces, specifically used for the knee, into four categories: prophylactic, rehabilitative, functional and patellofemoral. All provide stability, apply precise pressure, and/or help maintain alignment of the knee joint at set constants.

Some of the more innovative designs allow torsion to be applied at the knee joint and new technology has further improved their efficiency by allowing the torque to be adjusted. However, the lack of real-time abilities is a significant downside for these devices that limits their overall effectiveness in rehabilitation. The inclusion of active components has been a widely accepted method of improving upon this deficiency.

This seemingly small addition has considerable drawbacks though. The application of traditional active elements increases the overall size, cost, weight, and other related characteristics. Equally important are the concerns with control and sensory feedback, which would also be considered necessary with the addition of active components. All these combined, along with the obvious goals of making the systems as efficient and beneficial to an individual during rehabilitation as possible, force their designs to go beyond that of a portable orthosis, and more so a machine.

In terms of rehabilitation, the most effective methods known today are these rehabilitation machines. They are commonly used for rehabilitating and strengthening patients, subjects, and athletes while providing quantitative measurements of their performance. They provide high resistive and sometimes assistive forces, while providing a unique tailoring of the rehabilitation regime to nearly any individual. This ability dramatically increases their proficiency as a rehabilitation tool. Their services have been limited to primarily only physical therapy offices though, as a direct result of their shear size, weight, and cost.

Electro-rheological fluids (ERFs) are fluids that experience dramatic changes in rheological properties, such as viscosity, in the presence of an electric field. Willis M. Winslow first explained the effect in the 1940's using oil dispersions of fine powders [13]. The fluids are made from suspensions of an insulating base fluid and particles on the order of one tenth to one hundred microns (in size). The volume fraction of the particles is between 20% and 60%. The electro-rheological effect, sometimes called the Winslow effect, is thought to arise from the difference in the dielectric constants of the fluid and particles. In the presence of an electric field, the particles, due to an induced dipole moment, rearrange into a more organized manner, or form chains along the field lines. These chains alter the ERF's viscosity, yield stress, and other properties, allowing the ERF to change consistency from that of a liquid to something that is viscoelastic, such as a gel. ERF's generally respond to changes in electric fields in a matter of only a millisecond or two. Good reviews of the ERF phenomenon can be found in [14,15].

Control over a fluid's rheological properties offers the promise of many possibilities in engineering, especially actuation and control of mechanical motion. Devices that rely on hydraulics can benefit from ERF's quick response time and reduction in device complexity. Their solid-like property in the presence of a field can be used to transmit forces over a large range and have found a number of applications. A list of many engineering and practical applications of ERFs can be found in [16]. Our team has developed several prototypes of ERF-based linear and rotary actuation elements [17,18], which can apply controllable resistive forces and torques such as the Flat Plate (FP) rotary actuator concept which is the primary component of the ERF actuated knee orthosis described below.

### Design and Prototype

The ERF knee device possesses the ability to accurately provide large resistive forces with full real-time control while remaining completely portable and wearable. These characteristics make it an ideal apparatus for several applications. For active rehabilitation exercises it replaces the need for overly cumbersome and reasonably outdated machines, by remaining a lightweight portable system that is capable of all the same forces, control, and more. Similarly, it replaces the need for large weight-lifting machines. For gait-training purposes, such as in stroke patients with hyperextension difficulties, it is a viable clinical device. Through the sensors embedded in the device, computer closed-loop control, and clinical training these disabilities are overcome by providing real-time resistance that limits motion and supports the weight of the user, to retrain a proper gait. Additionally, the portability of the device adds a whole new dimension to rehabilitation and exercising in general, where the patient is now able to take a powerful isokinetic machine home, to work, on vacation, or wherever else they may travel.

**Figure 4:**
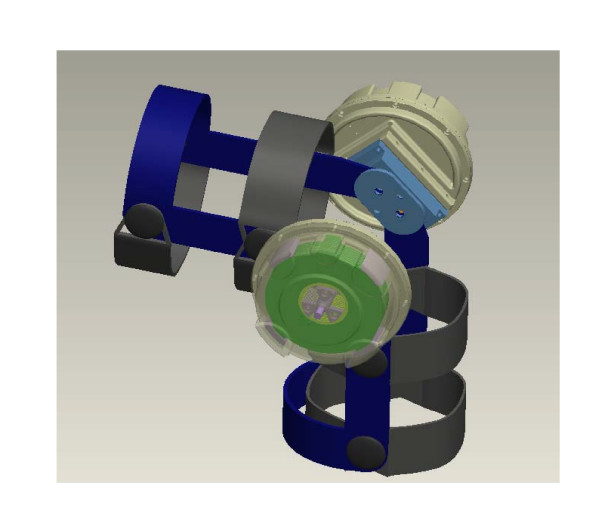
CAD rendering of electro-rheological fluid based knee orthosis.

The design of this innovative device consists of three major subsystems – an ERF based resistive actuator, a gear system, and the structural brace frame. The ERF based resistive actuators, which provide a bias force to the knee joint, simulating whatever forces desired, consist of multiple parallel rotating electrode plates and they are called Flat Plate resistive actuators. They are attached via a gear system to a standard brace as seen in the CAD rendering of Figure 4.

**Figure 5:**
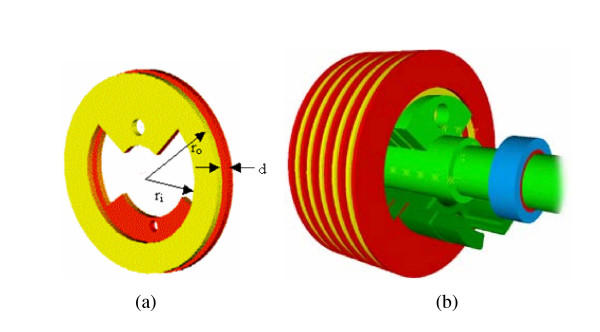
(a) Electrode plates (b) Internal assembly of the FP ERF resistive actuator.

Several circular copper plates (shown in Figs. 5a and 5b) are located parallel to each other, on a fixed axis. On a parallel, concentric axis, are another set of copper plates, which lie parallel and alternate with the fixed plates. The latter set of plates can rotate relative to the fixed plates, and the small gap between the plates contains ERF. Applying an electric field across the gap causes the fluid properties to change (in a matter of milliseconds), resulting in an increase in yield stress. The change physically alters the fluid from the consistency of thin oil to that of a thick gel. This property is used to control the resistive forces of the ERF FP actuator. The copper electrode plates with an inner and outer radius, r_i _and r_o_, respectively and a gap of d between plates can be seen in Figure 5a. Based upon the dimensions of the variables r_i_, r_o_, and d the design of the FP resistive actuator can be adjusted to produce a device capable of the resistive torques needed for any application. Figure 5b shows the assembly of a multiple Flat-Plate ERF element in CAD.

The entire ERF assembly is housed in a casing that seals in the fluid and attaches to a gearbox. The gearbox transmits and multiplies the torque output of the FP resistive actuators while supporting them on the frame. The brace frame is an off-the-shelf knee brace with all the features necessary for the device. It boasts a polycentric hinge, comfortable strapping method, and a lightweight, rigid frame. Included in the design were optical encoders for measuring angle, speed, and acceleration of the knee.

**Figure 6:**
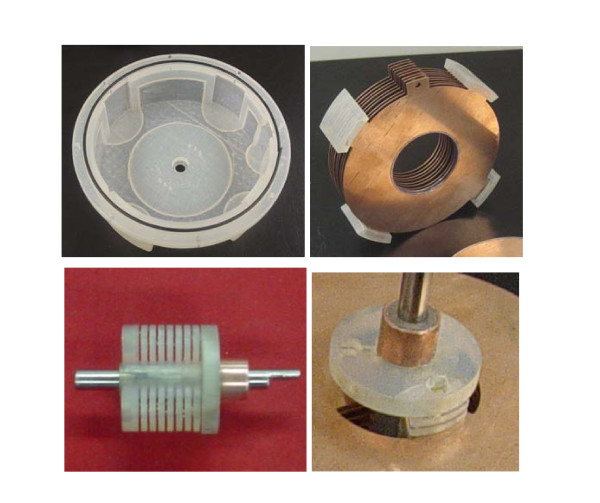
**Components of the brace's ERF FP actuator. **Fabricated case with o-ring seal (top left); CNC machined electrodes with rapid prototyped mounts (top right); fabricated rotating shaft with steel output shaft and commuter installed (bottom left); actuator shaft with rotating plates attached (bottom right).

**Figure 7:**
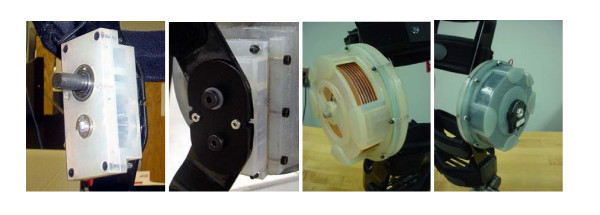
**Close up views of the ERF actuated brace. **Fabricated gearbox (left); Inner hinge (center left); Attached Actuator casing made with slots so inside plates are visible (center right); Fabricated actuator attached, filled with fluid, and encoder mounted (right).

**Figure 8:**
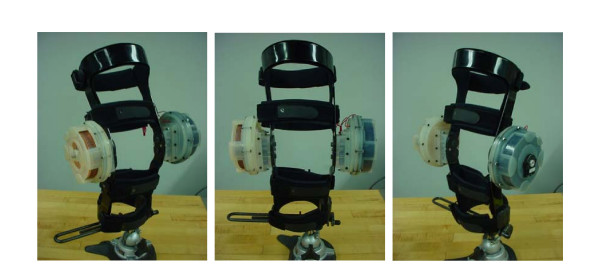
**First version prototype of the ERF driven knee rehabilitation orthosis. **Left actuator casing is made with slots so inside plates are visible, right actuator is filled with fluid.

An initial prototype of the design was built for feasibility analysis. The final actuator was rapid prototyped using a 3D Systems Viper 2000i^2 ^machine. It was bench tested and was found to produce a maximum resistive torque of 9.16 N·m. A Don Joy 4TITUDE™ knee brace was donated by the company Don Joy Orthopedics, slightly disassembled and machined to allow for attachment of the gearbox and actuator. A gear ratio of 1:1.67 was used resulting in an overall device resistance of approximately 30.16 N·m. The final system successfully demonstrated an accurate and easy controllable system for resisting knee motion. In Table 2 a summary of the device characteristics and the actuator parameters can be found. Below are several images of the prototype and close-ups of some of the individual parts (Figs. 6, 7, 8).

**Table 2:** ERF Based Knee Device Design Summary

**Actuator Parameters:**	
Gap size (d)	1.0 mm
Inner Radius (r_i_)	20.0 mm
Outer Radius (r_o_)	45 mm
Number of Plates	17
Actuation Voltage	4.25 kV
Maximum Actuator Torque	9.17 N·m
**System Characteristics:**	
Gear Ratio	1:1.67
Torque Produced by Device	30.16 N·m

**Figure 9:**
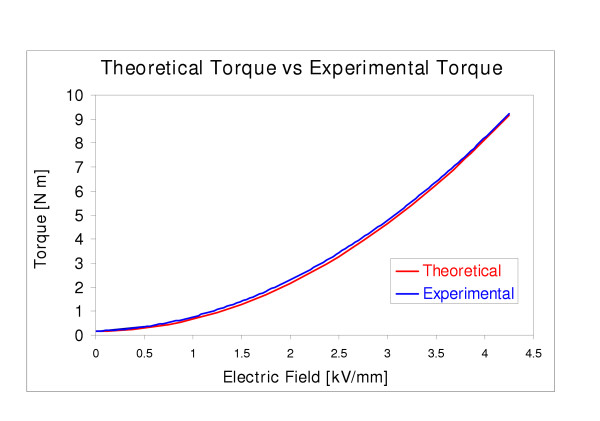
Theoretical vs. experimental torque of final designed/fabricated actuator for ERF driven knee rehabilitation orthosis.

So far tests were performed to verify the capabilities of the actuators. Since two identical actuators were used, the verification of one of these actuators would be theoretically as accurate as creating a duplicate of a human knee joint for the purpose of testing the whole device. The average torque output of the actuator at each voltage was plotted and compared to the predicted theoretical equation's results. Figure 9 was the result and the two plots show a very close resemblance. The accurate results therefore suggest that the proposed system is capable of the forces desired.

## Electrical stimulation and biofeedback knee device

### Overview

A knee brace that can be used in multiple stages of rehabilitation by using various therapy techniques was developed by our team. Following knee surgery most patients experience muscle atrophy and in some cases nerve damage. To overcome these problems physical therapists have turned to the use of electrical stimulation (E-Stim) and biofeedback (EMG) as the preferred methods of treatment. These forms of therapy help to increase the range of motion of the knee and improve neuromuscular re-education. By incorporating these units, along with a rotary encoder, into a post-operative brace it is possible to monitor the progress of the patient in a unified computer controlled setting. It also allows the patient to perform the rehabilitation while walking in a stable brace which promotes proper gait. A graphical user interface was built in LabView to monitor the various sensing units of the biofeedback knee brace.

### Significance and Background

During physical therapy, an articulated controlled motion and exercise is crucial in a successful physical therapy process. Controlled motion benefits the ligaments, bones, and soft tissue and prevents them from becoming degenerative. Resistance exercises help build muscle mass and restore functionality to the limbs. Devices such as the two previously mentioned are ideal for these two cases. Alternative systems exist however, that use very different methods for overcoming the same aspects in rehabilitation. These include electronic stimulation and biofeedback.

Electrical Stimulation (E-Stim) is a rehabilitative treatment that stimulates nerves by sending an electrical current through the skin. In knee surgery rehabilitation the E-Stim is typically used to activate the muscles around the knee for the purposes of neuromuscular re-education. In the early post-operative stages the E-Stim is used for active rehabilitation, where it stimulates the motor nerves of muscles without the patient's effort. In the secondary stages of therapy the E-Stim is used in active-assisted motion, where patient uses their muscles along with the external stimulation to move the joint. E-Stim can also be used while exercising muscles as well.

Biofeedback implemented by EMG is a device that monitors muscle activity. The feedback provides valuable information regarding progress and muscle performance. The data from this device allows therapists to gain a better understanding of how the patient is responding to the treatment. This means that the therapy can be better tailored to the individual.

Following knee surgery many patients experience muscle atrophy and in some cases nerve damage. To overcome these problems, physical therapists have turned to the use of electrical stimulation and biofeedback as the preferred methods of treatment. These forms of therapy help to increase the knee range of motion as well as reduce pain, swelling, and total recovery time.

### Design and Prototype

**Figure 10:**
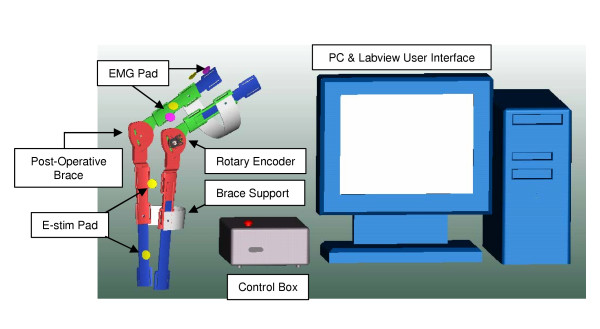
Schematic of the smart knee brace.

**Figure 11:**
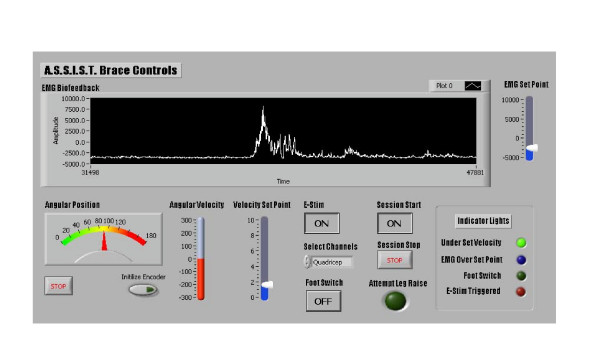
Graphical controls interface for the electrical stimulation and biofeedback knee device.

A knee brace combining electrical stimulation with the sensory information from biofeedback and other sensors has been developed as shown schematically in Figure 10. The information from the biofeedback and a rotary encoder are fed to a computer. The computer compares the EMG information to the data it receives from the linear encoder. If it detects a bio-signal being sent from the brain to the leg, but there is no motion in the brace, the E-Stim is triggered to assist the patient. All information gathered by the sensors is presented to the operator within a graphical interface, where it is also possible to adjust the settings for proper customizing of the exercises (see Figure 11 for a screen capture of this interface).

The flexibility and computer control of this device results in a valuable autonomous tool for a variety of rehabilitation exercises. The system, as described, can be used in place of or in conjunction with any exercise involving passive rehabilitation or active-assisted (the two earlier post-operative rehabilitation stages). With the addition of the foot switch, which is placed under the sole of the patient's shoe, the system can be used to aid in walking and regaining proper mobility. When pressure is exerted on the switch, it triggers the electrical stimulation on the quadriceps as well as the tibialis anterior, to assist in such situations as with those who suffer from foot drop due to nerve damage. Furthermore, the system boasts the added benefits of allowing the patient to perform the walking exercises while wearing a stable brace (promoting a more proper gait) and establishes an easy way to monitor their progress.

**Figure 12:**
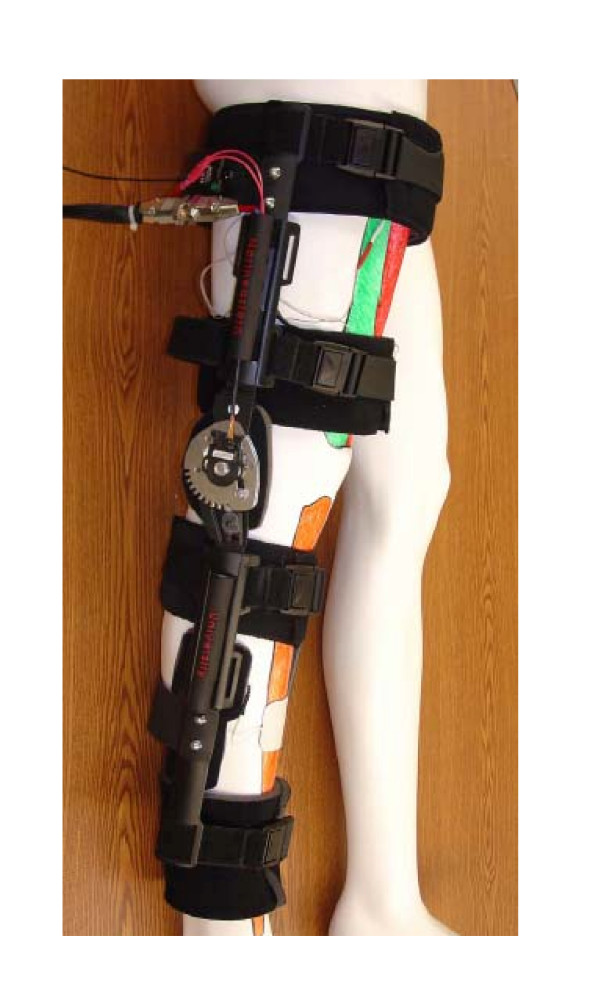
Electrical stimulation and biofeedback knee device.

**Figure 13:**
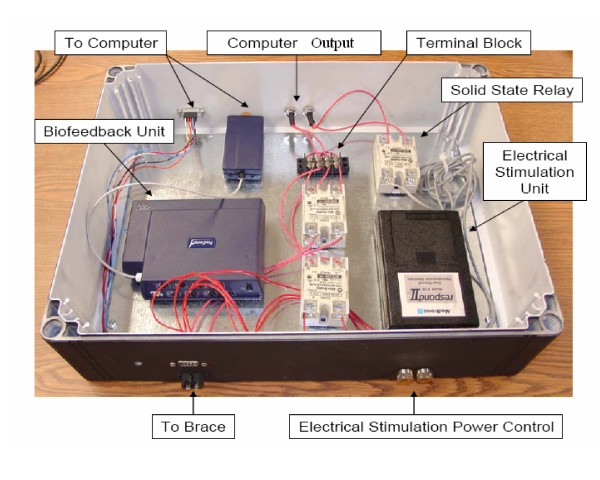
Control box for the electrical stimulation and biofeedbackdevice.

A prototype was developed to demonstrate the proposed concept as shown in Figure 12. This prototype uses a Don Joy TROM knee brace as a frame for the device. The padding for this brace was adapted to allow the e-stim and EMG electrodes to be adhered to the proper locations on the leg. Polyethylene brace supports were also added to the upper and lower sections of the brace. This adds stability and makes the brace significantly easier to put on. In addition, the hinge on the brace was modified so that a 1/4" diameter shaft rotates with the lower section of the brace. A Renco rotary encoder was attached to this shaft so that the angle and velocity of the knee brace could be determined. The wiring from the e-stim, EMG and the rotary encoder is covered by a custom wiring conduits located along the rails of the brace. Once the wiring leaves the brace it is hooked up to a control box, which houses all of the electronics. This includes the ProComp Infiniti biofeedback unit, the Respond II e-stim unit, as well as two solid state relays. This control box is also connected to a personal computer. Figure 13 is a picture of the control box with the components labeled.

A graphical user interface in Labview is responsible for controlling the response of the system (see Figure 11). The information from the EMG biofeedback and the rotary encoder are fed to a computer via Labview. The computer will then compare the EMG information to the data it receives from the rotary encoder. If it detects a signal being sent from the brain to the leg, but there is no motion in the brace, the e-stim will be triggered to assist the patient. This will help re-educate the neuro-muscular system. This information will be presented to the operator with a graphic interface. By controlling the operation of the e-stim through adjustable set points in the lab view program the physical therapist will be able to apply the device throughout the entire rehabilitation process.

**Figure 14:**
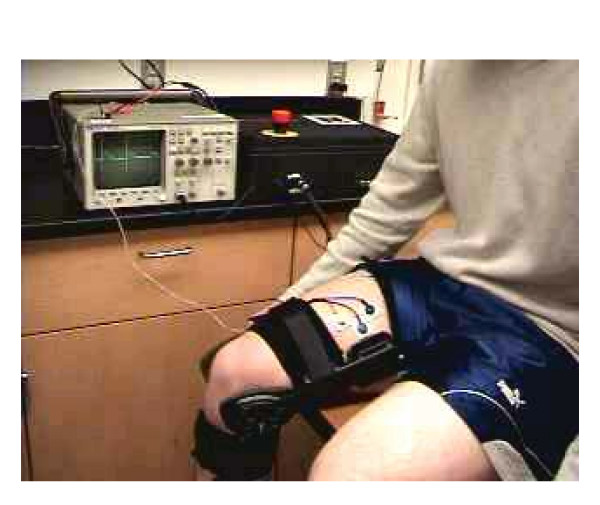
Human subject wearing the instrumented biofeedback knee device.

**Figure 15:**
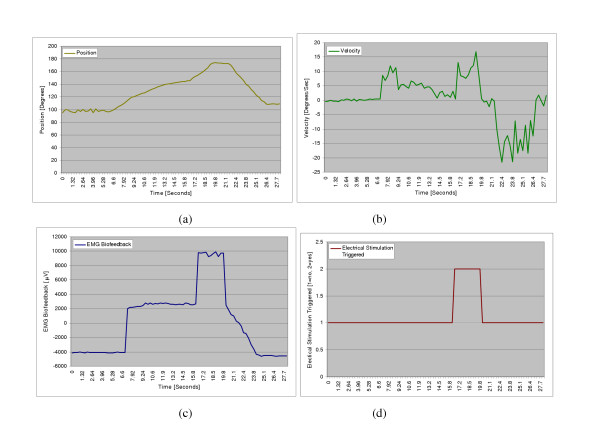
**Test on quadriceps muscles (extension). **(a) Position; (b) Velocity; (c) EMG Biofeedback; (d) Electrical stimulation.

The device was tested to ensure all the components were coordinated correctly. The test subject was instructed to perform a steady extension of the knee as shown in Figure 14. In the testing, the subject attempts to do seated leg extensions with the help of the brace. In this exercise the subject sits on the edge of a table and attempts to extend their lower leg horizontally. When the subject reaches a point where they can't extend their leg any further under their own power, the EMG biofeedback senses this data. Then the logic in the controls will compare the readings with the information from the rotary encoder. If the EMG signals are above the threshold this is set and the rotary encoder is stationary then the e-stim is triggered. The e-stim causes the quadriceps to contract helping the subject to achieve full extension. Since we were testing on a healthy subject, the threshold was set to a low value so that the e-stim was triggered in the middle of the exercise. Figs. 15a–d shows the results of one of these tests. Once reaching his maximum point, the EMG unit is seen detecting the muscle trying to move with no movement registered by the encoder. This triggered the E-Stim device and current was sent to the muscle, forcing the muscle to complete the extension movement. All processes worked correctly setting the ground work for a very versatile and efficient system.

## Conclusion

The designs of the three systems described involve the meshing of standard, mechanical solutions and novel, new age ones. Besides being innovative the devices were all designed with a few key factors in mind. Compactness and portability were desired to compete with present day equipment that is currently quite large, permanent, and tedious to set-up and use. This was obviously met in each case, where all three devices are wearable, can be transported easily and involve computer control that simplifies their use significantly. Finally, the prototypes of portable rehabilitation devices presented here did demonstrate that these concepts are capable of the performance their commercially available but non-portable counterparts exhibit. After proving this feasibility, analyzing the efficiency of these devices is the next obvious phase.

All three devices will be undergoing redesigning and construction to optimize their operation in order to proceed to human testing. Collaborations with large companies have been established for the development of specialized braces for these projects. Additionally, collaborations and arrangements are already in place for human testing at Spaulding Rehabilitation Hospital located in Boston, Massachusetts. If these tests are successful, they will open the door to a new era in rehabilitation where the recovery process could be dramatically improved through the use of a whole new breed of rehabilitation devices.

## References

[B1] Quintinskie J Shoulder Rotor Cuff and Elbow Stretching Machine. US Patent 6,007,500.

[B2] Blauth W, Knull E Apparatus for Postoperative Exercising of Elbow and Shoulder Joints. US Patent 4,669,451.

[B3] Mauldin D, Jones R Elbow Device. US Patent 4,433,679.

[B4] Pape L Elbow Brace. US Patent 6,530,868.

[B5] Hotchkiss R, Hotchkiss K, Woodward A Dynamic Elbow Support. US Patent 5,102,411.

[B6] Carlson D Portable Controllable Fluid Rehabilitation Devices. US Patent 5,711,746.

[B7] Biodex Medical Systems, Inc., Biodex System 3. http://www.biodex.com/rehab/system3/system3_feat.htm.

[B8] Advanced Brace, Catalog of Braces and Support. http://ankle-foot.com/orthoplex/breg/breg/post-op-elbow.htm.

[B9] Dyna Splint Systems, INC. http://www.dynasplint.com/products_frame.html.

[B10] Medical Technology INC., Bledsoe Brace Systems. http://bledsoebrace.com/products/arm/ext_arm.htm.

[B11] Ultraflex Systems, INC., EO Custom Molded. http://www.ultraflexsystems.com/bracedesigns/eocm.htm.

[B12] Orthomerica, Prime Elbow System. http://www.orthomerica.com/products/upext/prime_elbow.htm.

[B13] Winslow WM (1949). Induced fibrillation of suspensions. Journal of Applied Physics.

[B14] Block H, Kelly JP (1998). Electro-rheology. Journal of Physics D: Applied Physics.

[B15] Gast AP, Zukoski CF (1989). Electro-rheological fluids as colloidal suspensions. Advances in Colloid and Interface Science.

[B16] Mavroidis C (2002). Development of advanced actuators using shape memory alloys and electro-rheological Fluids. Research for Non-Destructive Evaluation.

[B17] Mavroidis C, Bar-Cohen Y, Bouzit M, Bar-Cohen Y (2001). Chapter 19: Haptic interfaces using electro-rheological fluids. Electroactive Polymer (EAP) Actuators as Artificial Muscles: Reality, Potentials and Challenges.

[B18] Melli-Huber J, Weinberg B, Fisch A, Nikitczuk J, Mavroidis C, Wampler C (2003). Electro-rheological fluidic actuators for haptic vehicular instrument controls. Proceedings of the Eleventh Symposium on Haptic Interfaces for Virtual Environment and Teleoperator Systems: 22–23 March 2003; Los Angeles.

